# Species Diversity in the Parasitoid Genus *Asobara* (Hymenoptera: Braconidae) from the Native Area of the Fruit Fly Pest *Drosophila suzukii* (Diptera: Drosophilidae)

**DOI:** 10.1371/journal.pone.0147382

**Published:** 2016-02-03

**Authors:** Emilio Guerrieri, Massimo Giorgini, Pasquale Cascone, Simona Carpenito, Cees van Achterberg

**Affiliations:** 1 Institute for Sustainable Plant Protection, National Research Council of Italy, 80055, Portici (Na), Italy; 2 Naturalis Biodiversity Center, 2300 RA, Leiden, Netherlands; 3 Department of Life Sciences, The Natural History Museum, London, SW7 5BD, United Kingdom; Institute of Zoology, CHINA

## Abstract

*Drosophila suzukii* (Matsumura), commonly known as Spotted Wing Drosophila (SWD), is a worldwide serious economic threat to the production of berries and stone fruits. The chemical control widely used against this pest is often not able to preventing yield losses because wild flora offers an abundance of fruits to *D*. *suzukii* where the pest is able to reproduce and from where it recolonizes neighbouring cultivated fields. Alternatively, within Integrated Pest Management protocols for *D*. *suzukii*, biological control could play a key role by reducing its populations particularly in non-cultivated habitats, thus increasing the effectiveness and reducing the side negative effects of other management strategies. Because of the scarcity and of the low efficiency of autochthonous parasitoids in the new invaded territories, in the last few years, a number of surveys started in the native area of *D*. *suzukii* to find parasitoid species to be evaluated in quarantine structures and eventually released in the field, following a classical biological control approach. This paper reports the results of these surveys carried out in South Korea and for the first time in China. Among the parasitoids collected, those belonging to the genus *Asobara* Foerster resulted dominant both by number and species diversity. By combining morphological characters and the mitochondrial COI gene as a molecular marker, we identified seven species of *Asobara*, of which two associated with *D*. *suzukii*, namely *A*. *japonica* and *A leveri*, and five new to science, namely *Asobara brevicauda*, *A*. *elongata*, *A mesocauda*, *A unicolorata*, *A*. *triangulata*. Our findings offer new opportunity to find effective parasitoids to be introduced in classical biological control programmes in the territories recently invaded by *D*. *suzukii*.

## Introduction

*Drosophila suzukii* (Matsumura), commonly known as Spotted Wing Drosophila (SWD), is a worldwide serious economic threat to the production of berries and stone fruits [1, 2]. This pest, native of the South-eastern Asia, has been reported simultaneously from Europe and North America in 2008 quickly becoming a key pest of soft and thin skin fruit crops in many locations [3,4,5,6]. More recently new records of the pest include Central and South America [7,8]. The management of SWD in fruit production areas invaded by this pest mainly relays on pesticides [6]. However, this approach is often ineffective in preventing yield losses because wild flora (and fruits) neighbouring the cultivated fields offers a valid alternative for reproduction and refuge sites from where recolonize the crops [9]. Moreover, there is a growing concern about the negative side-effects of pesticides on the environment and for the possible selection of resistant populations. Alternative strategies for controlling this pest are highly desirable and among these, biological control could play a key role particularly in reducing *D*. *suzukii* populations in non-cultivated habitats, with positive effects on other management strategies applied in field crops.

To date, field surveys and laboratory tests suggested that only few parasitoid and predator species attacking Drosophilids in Europe and North America adapted to the invasive *D*. *suzukii*, although they resulted ineffective inreducing its populations below economic damage thresholds [10–13]. The absence of endemic effective biocontrol agents in Europe and North America prompted the initiation of surveys in the native range of *D*. *suzukii* to find parasitoid species to be evaluated in quarantine and eventually released in the field, following a classical biological control approach. In this work, within the framework of an international project funded by the European Union aiming at enhancing the sustainable control of invasive pests, a survey of parasitoids of *D*. *suzukii* has been carried out in China and South Korea, for which no record is available. Among the parasitoids collected, those belonging to the genus *Asobara* Foerster were dominant both by number and species diversity. By combining a morphological and a molecular approach, we identified seven species of *Asobara* including five new to science and two associated with *D*. *suzukii*. Our findings offer new opportunity to find effective parasitoid/s to be introduced in classical biological control programmes in all countries recently invaded by *D*. *suzukii*.

## Material and Methods

### Insect collection

Sampling of *D*. *suzukii* and other Drosophilids parasitoids has been carried out in different locations of China (Yunnan Province) and South Korea (see material examined for detailed locations) considered to be part of the native area of *D*. *suzukii*. Both unspoiled wild areas and cultivated fields (mainly blueberry but also pear, peach and Chinese bayberry -*Myrica rubra*-cultivations) were surveyed. In each location two strategies of collection were adopted. The first one was harvesting soft fruits from trees and shrubs (e.g. blueberries, blackberries and Chinese bayberries) and storing them in aerated boxes in a climatic chamber at 25°C. All boxes were checked daily for parasitoid (and fly) emergence for 3 weeks. The second strategy consisted in positioning fruit-baited traps in the field prepared by cutting a few holes (0.5 mm diameter) on air tight plastic boxes commonly used for preserving food in the fridge (30x15x10cm). Each box was filled with sliced fruits (mainly banana, melon) either uninfested or previously exposed for a few days to hundreds mated females of *D*. *suzukii*. A transect of ten fruit-baited traps 100 m away from each other was composed in wild vegetation or in cultivated blueberry fields. Traps were collected one week after set up and parasitoids found crawling in the traps on fruit slices were collected. All traps were kept at 25°C in a climatic chamber (the holes and the lid covered with fine mesh gauze) and checked daily for the emergence of parasitoids (and flies) for 3 weeks. Each parasitoid collected was readily killed in absolute ethanol and preserved at -20°C.

No specific permissions were required for all locations/activities that were conducted with colleagues of local Institutions (see Acknowledgements section). As for private farms we were invited by owners to carry out investigations for reducing the impact of *Drosophila suzukii* in their propereties. Human participants, specimens or tissue samples, or vertebrate animals, embryos or tissues were not involved in this research. No endangered or protected species were involved in this research.

### Morphological analysis

For card mounting, insects stored in absolute ethanol were dried chemically by placing them in a 1:1 absolute ethanol:xylene solution; after 24 hours they were transferred into amyl acetate for 24 hours and then rinsed in amyl acetate until its complete evaporation. They were then mounted on card using a water soluble glue. For slide mounting the protocol reported by Noyes has been followed [14]. In brief, wings were mounted in Canada balsam without processing while the remaining parts were kept for 5 minutes in a 10% KOH solution at 100°C, for 5 minutes in acetic acid and dehydrated in ethanol from 70% to 100%. A drop of clover oil was added to absolute ethanol and after alcohol evaporation the insect was dissected and mounted in Canada balsam. The slides were left for 2 hours on a hotplate to harden the balm before positioning the coverslips. All descriptions and photographs were made using a dissection microscope (LeicaZ16 APO microscope equipped with a Leica DFC 425C digital camera).

Morphological terminology used for species description follows van Achtherberg [15,16]. A new record of distribution is indicated by an asterisk (*) preceding the country.

The studied material will be deposited in the following depositories:Institute of Zoology, Chinese Academy of Sciences, Beijing, China (IZCAS) and Naturalis Biodiversity Center, Leiden, Netherlands (RMNH including collections from former National Museum of Natural History, Leiden, the Entomological Institute, Wageningen and the Zoological Museum, Amsterdam).

### Molecular analysis

Before card mounting and after their attribution to the genus *Asobara* and to a specific morphospecies, whole specimens (see Table 1 for the complete list of samples examined) were subjected to genomic DNA extraction following a non-destructive protocol using a Chelex-proteinase K-based without grounding the specimen [17]. Partial sequence of the mitochondrial *cytochrome oxidase subunit I* gene (COI) was determined for each studied individual and the phylogenetic relationships investigated.

**Table 1:** *Asobara* specimens used for molecular characterization.

Species	Localities	Voucher	Collection date	Accession N°
*A*. *unicolorata*				
	Yunnan-China	DSZ083	24/07/2013	KT835409
	Yunnan-China	DSZ055	23/07/2013	KT835410
	Yunnan-China	DSZ053	24/07/2013	KT835411
	Yunnan-China	DSZ006	24/07/2013	KT835412
*A*. *triangulata*				
	Yunnan-China	DSZ062	24/07/2013	KT835413
*A*. *mesocauda*				
	Yunnan-China	DSZ061	24/07/2013	KT835414
	Yunnan-China	DSZ060	24/07/2013	KT835415
	Yunnan-China	DSZ059	22/07/2013	KT835416
	Yunnan-China	DSZ058	22/07/2013	KT835417
	Yunnan-China	DSZ056	23/07/2013	KT835418
	Yunnan-China	DSZ052	23/07/2013	KT835419
	Yunnan-China	DSZ051	23/07/2013	KT835420
	Yunnan-China	DSZ049	23/07/2013	KT835421
	Yunnan-China	DSZ014	23/07/2013	KT835422
	Yunnan-China	DSZ013	23/07/2013	KT835423
	Yunnan-China	DSZ012	24/07/2013	KT835424
	Yunnan-China	DSZ011	24/07/2013	KT835425
	Yunnan-China	DSZ010	24/07/2013	KT835426
*A*. *leveri*				
	South Korea	DSZ084	05/07/2013	KT835427
	South Korea	DSZ023	04/08/2013	KT835428
	South Korea	DSZ022	04/08/2013	KT835429
	South Korea	DSZ021	04/08/2013	KT835430
	South Korea	DSZ020	04/08/2013	KT835431
	South Korea	DSZ016	04/08/2013	KT835432
*A*. *japonica*				
	South Korea	DSZ082	07/07/2013	KT835433
	South Korea	DSZ081	12/07/2014	KT835434
	South Korea	DSZ080	12/07/2014	KT835435
	South Korea	DSZ079	11/08/2013	KT835436
	South Korea	DSZ078	11/08/2013	KT835437
	South Korea	DSZ077	11/08/2013	KT835438
	South Korea	DSZ076	11/08/2013	KT835439
	South Korea	DSZ074	08/07/2014	KT835441
	South Korea	DSZ073	08/07/2014	KT835442
	South Korea	DSZ072	09/07/2014	KT835443
	South Korea	DSZ071	12/07/2014	KT835444
	South Korea	DSZ070	12/07/2014	KT835445
	South Korea	DSZ069	12/07/2014	KT835446
	South Korea	DSZ068	12/07/2014	KT835447
	South Korea	DSZ067	12/07/2014	KT835448
	South Korea	DSZ024	11/08/2013	KT835449
	South Korea	DSZ018	01/07/2013	KT835450
	South Korea	DSZ017	01/07/2013	KT835451
*A*. *elongata*				
	Yunnan-China	DSZ048	23/07/2013	KT835452
*A*. *brevicauda*				
	South Korea	DSZ066	05/07/2013	KT835453

COI was amplified using one of the following primer combinations: LCO and HCO [18], LepF1 and LepR1 [19] or LCOasob and HCO [20]. In a few cases a semi-nested PCR was conducted using one of the above mentioned forward primers and the reverse primer HCOout [21] followed by one of the combinations above cited as second amplification. Reactions were performed in 15 μl volumes containing: 3μl of5 X GoTaq buffer (Promega); 1.5 μl dNTP (2.5 mM each); 0,75 μl of forward and reverse primer (10 μM each); 0.2 μl GoTaq^™^ DNA Polymerase (5u/μl) and 1μl template DNA. Amplifications were achieved using a Biorad thermocycler Mycycler (Biorad) programmed for: 1 min at 94°C; followed by 40 cycles of 30 s at 94°C, 90 s at 48°C, and 60 s at 72°C; and a final step of 7 min at 72°C. PCR products were visualized after electrophoresis on 1.5% agarose gel stained with ethidium bromide to confirm the amplification. Fragments obtained were sequenced in both sense and antisense directions by adopting EZ-seq standard service (Macrogen Inc., Seoul, Korea). Sequence alignment were performed in BioEdit 7.0 [22] with ClustalW tool and edited manually. COI sequences were verified for protein coding frameshifts and nonsense codons using MEGA6 [23]. COI sequences of *Asobara* individuals were deposited in GenBank under the accession numbers KT835409-KT835453.

Phylogenetic and molecular evolutionary analysis were conducted using MEGA 6 [23]. Phylogeny was constructed using maximum likelihood (ML) and neighbor-joining (NJ) analysis. ML tree was obtained implementing the GTR+G substitution model, selected in MEGA6 as the better to describe the substitution pattern (lowest BIC score), and five discrete gamma categories. Missing data were treated with the pairwise-deletion option and all codon positions were included in the analysis. For tree inference, the nearest-neighbor-interchange (NNI) heuristic method was used and the initial tree selected using the NJ/BioNJ option. ML branch support was based on 1,000 bootstrap replications. NJ tree was constructed using the Tamura 3-parameter method to compute the evolutionary distances, treating missing data with the pairwise-deletion option and running 1,000 bootstrap replications for branch supporting. ML and NJ analyses involved 91 nucleotide sequences and a total of 711 positions in the final dataset. In addition to 44 COI sequences produced in this work, 45 *Asobara* COI sequences were retrieved from GenBank. These latter included sequences of *Asobara citri* (Fisher), *A*. *leveri* (Nixon), *A*. *japonica* Belokobylskij, *A*. *persimilis* (Papp), *A*. *pleuralis* (Ashmead), *A*. *rossica* Belokobylskij, *A*. *rufescens* (Foerster), *A*. *tabida* (Nees von Esenbeck), and sequences of undescribed *Asobara* spp. from Japan. Further, COI sequences of *Rhygoplitis terminalis* (Gahan) and *Apanteles anarsiae* Ashmead were recovered from GenBank and used as outgroups in phylogenetic analyses. Uncorrected intra- and interspecific *p*-distances (number of base differences per site) based on COI sequences were also calculated using MEGA6.

### Nomenclatural acts

The electronic edition of this article conforms to the requirements of the amended International Code of Zoological Nomenclature, and hence the new names contained herein are available under that Code from the electronic edition of this article. This published work and the nomenclatural acts it contains have been registered in ZooBank, the online registration system for the ICZN. The ZooBank LSIDs (Life Science Identifiers) can be resolved and the associated information viewed through any standard web browser by appending the LSID to the prefix “http://zoobank.org/”. The LSID for this publication is: urn:lsid:zoobank.org:pub:AAF73644-AE86-4546-8D20-AA3A870DCD48. The electronic edition of this work was published in a journal with an ISSN, and has been archived and is available from the following digital repositories: PubMed Central, LOCKSS [author to insert any additional repositories].

## Results

The vast majority of the parasitoids collected from fruit-baited traps and ripe fruits belonged to the genus *Asobara* Foerster. Morphological analysis allowed the identification of discrete characters able to group the specimens analysed in seven morphospecies. Two morphospecies, found only in South Korea, were recognised as *A*. *japonica* and *A*. *leveri*. The other five morphospecies, four from Yunnan and one from South Korea, turned out to be undescribed. The identity of the seven morphospecies was substantiated by results of molecular analysis, leading us to describe five new species (namely *A*. *elongata*, *A*. *mesocauda*, *A*. *unicolorata* and *A*. *triangulata*)from China and one (*A*. *brevicauda*)from South Korea and to provide a morphological key for the identification of the seven *Asobara* species.

### Morphological analysis

#### Genus *Asobara* Foerster, 1863. Synonymy

*Spanista* Foerster, 1863. Wharton (1980) included *Sathra* Foerster, 1863 [24], but this is a synonym of *Phaenocarpa* Foerster, 1863.

#### Diagnosis

Third article of antenna distinctly shorter than fourth article; first subdiscal cell of fore wing widely open because of absence of veins CU1b and 2-1A; maxillary palp with 5–6 articles; veins 2-SR and 1-SR+M of fore wing present; vein cu-a of hind wing of macropterous specimens variable, if absent then lateral carina of mesoscutum absent in front of tegulae and face without a pair of medium-sized vertical grooves above clypeus.

#### Biology

Rather large genus, contains parasitoids of Drosophilidae and Sepsidae in decaying organic matter, especially fruits and leaves. Species with widened ovipositor sheath have been reared as parasitoids of Tephritidae in fruits.

#### Distribution

Cosmopolitan [25].

#### Key to *Asobara* species from China and South Korea Females

1. Setose part of ovipositor sheath 0.4x as long as hind tibia ……… ***A*. *brevicauda* sp. nov.**
  - Setose part of ovipositor sheath at least about as long as hind tibia …………………. 2
2. Mesoscutum without medio-posterior pit; precoxal sulcus smooth or nearly so ……………………………………………………3
  - Mesoscutum with medio-posterior pit; precoxal sulcus finely crenulate medially, rarely smooth …………………………………………………………………………6
3. Face dark brown, strongly contrasting with yellowish clypeus; first discal cell of fore wing rather robust; setose part of ovipositor sheath 1.1–1.2 times as long as hind tibia ………………………………………………………………………… 4
  - Face largely or completely brownish-yellow, similar to colour of clypeus; first discal cell of fore wing more elongate; setose part of ovipositor sheath about as long as hind tibia 5
4. Fourth antennal article strongly elongate about 10 times as long as wide; propodeum black ………………………………………………… ***A*. *elongata* sp. nov.**
  - Fourth antennal article not more than 7 times as long as wide; propodeum yellowish brown or reddish, rarely infuscate …………….. ***A*. *japonica*** Belokobylskij, 1998
5. 3-SR vein of fore wing about 5x as long as r vein ……………… ***A*. *mesocauda* sp. nov.**
  - 3-SR vein of fore wing about 6x as long as r vein ……………… ***A*. *triangulata* sp. nov.**
6. Second submarginal cell of fore wing narrow basally; medio-posterior pit of mesoscutum short elliptical ***A*. *leveri*** (Nixon, 1939)
  - Second submarginal cell of fore wing wide basally; medio-posterior pit of mesoscutum round or nearly so (Fig. 26) …………………………………………………… 7
7. Hind femur comparatively robust and widened subapically; vertex yellowish-brown; second submarginal cell of fore wing comparatively robust ***A*. *pleuralis*** (Ashmead, 1905)
  - Hind femur slender and parallel-sided subapically; vertex chestnut brown; second submarginal cell of fore wing comparatively elongate ……… ***A*. *unicolorata* sp. nov.**

**Fig 1:**
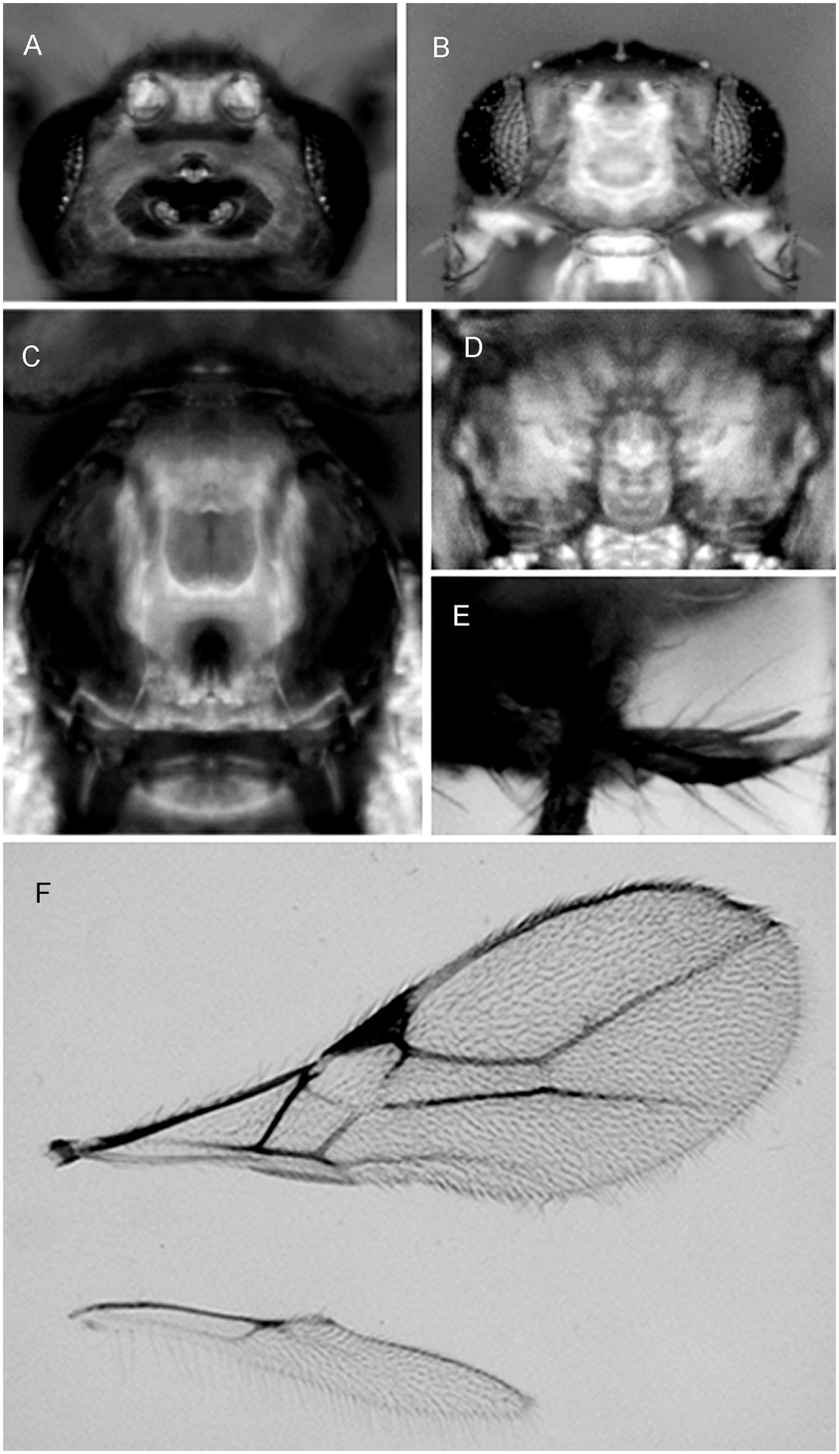
*Asobara brevicauda* sp. nov. ♀(A) Head in dorsal view. (B) Head in frontal view. (C) Mesoscutum in dorsal view. (D) Areola. (E) Ovipositor sheath. (F) Wings.

***Asobara brevicauda* van Achterberg and Guerrieri, sp. nov.** urn:lsid:zoobank.org:act:CE6AEC21-FCE3-4FAE-9793-62AAA06794D0 (Fig 1)

#### Material examined

**Type material**. Holotype, ♀, laboratory rearing from material collected in **South Korea**, Jirisan Park (35.22.51.451N 127.26.43.125E) 29.vii.2013, banana trap n.7 (EGKUZ2T72)(Guerrieri, Giorgini, Choi). Paratypes: 1♀ same data as holotype (EGKUZ2T71), 2♀, laboratory rearing from material collected in South Korea, Manhaengsan (little artificial lake with temple)(35.32.02.456N 127.26.13.929E), 29.vii.2013, banana trap n. 17 (EGKUZ3T175; EGKUZ3T176) (Guerrieri, Giorgini, Choi); 1♀, laboratory rearing from material collected in South Korea, Mudeungsan Park (path from cabin car to Baramajae Pass)(35.08.518N 126.59.034E), 30.vii.2013, banana trap n. 1, (Guerrieri, Giorgini, Choi). Holotype and paratypes will be deposited at RMNH.

**Non-type material:** 2♀, South Korea, Poyeongsa Jeollabuk-do (35°31'51.09''N 127°36'3.61''E), 5.vii.2015, ex Drosophilidae on berries of wild *Rubus* sp. (EGKUZ2T71- Dsz066- COI sequence KT835453) (J.Miller, B.Miller, H. Reidl, Y.Song)

Holotype, length of body (excluding ovipositor) 1.9 mm, length of fore wing 2.1 mm.

Head. Width of head 1.4 times its median length (Fig 1A and 1B), largely glabrous and strongly shiny dorsally; antenna with 23 articles, densely erect bristly setose, length of third article 0.8 times as long as fourth article, slender, length of third, fourth and penultimate articles 4.5, 6.5 and 3.0 times their width, respectively; length of maxillary palp 1.2 times height of head; eye in dorsal view (Fig 1A) twice as long as temple, sparsely setose; temple in dorsal view subparallel-sided; OOL:diameter of ocellus:POL = 6:2:3 (Fig 1A); minimum width of face 0.55 times maximum width of head and 0.75 times its height, smooth, with some long and erect setae; clypeus transverse and oval, its surface smooth and flattened; height of clypeus in lateral view equal to width of middle tooth of mandible (Fig 1B); anterior tentorial pits indistinct (Fig 1B); length of malar space 0.14 times basal width of mandible; mandible twice as long as wide, largely smooth, with a lamella ventrally and dorsally, with medium-sized carina connected to third tooth (Fig 1B).

Mesosoma. Length of mesosoma 1.4 times its height; pronotum without pronope; antescutal depression distinct and finely crenulate laterally; side of pronotum smooth; epicnemial area smooth; precoxal sulcus complete; remainder of mesopleuron smooth; episternal scrobe elliptical; pleural sulcus smooth, except for some crenulae medially and ventrally; mesosternal sulcus wide and with 2 short crenulae; metapleuron largely smooth, except for a few carinae ventrally; notauli only anteriorly impressed, with strong lateral carina and largely absent on disc; medio-posterior depression of mesoscutum small, deep and circular (Fig 1C); mesoscutum smooth and largely glabrous and its lateral carina interrupted in front of tegulae; scutellar sulcus moderately deep and with one carina, 0.4 times as long as scutellum; scutellum in lateral view rather flat; metanotum slightly lamelliform protruding dorsally in lateral view; surface of propodeum smooth except for a medium-sized median carina, rather short lamelliform costulae and a narrow parallel-sided areola (Fig 1D), its dorsal corners hardly protruding in lateral view and lateral carina of propodeum wide.

Wings. Pterostigma narrow triangular (Fig 1F); vein r about 0.5 times width of pterostigma, issued at apical 0.3 of pterostigma and vertical; r:3-SR:SR1 = 7:60:125; SR1 straight; cu-a obsolescent, postfurcal; 1-CU1:2-CU1 = 1:25; 2-SR:3-SR:r-m = 25:60:11; m-cu far antefurcal (2-SR+M 0.3 times as long as m-cu), forming a straight line with 2-SR+M, strongly converging to 1-M. Hind wing: M+CU + 1-M:1r-m = 60:107/16; cu-a absent.

Legs. Hind coxa smooth and elongate, rounded basally; tarsal claws slender medially; length of femur, tibia and basitarsus of hind leg 4.6, 10.0 and 5.0 times their width, respectively; hind femur, tibia and tarsus with long erect setae.

Metasoma. Length of first tergite 2 times its apical width, its surface largely smooth but coarsely rugose medially and laterally lamelliform, its dorsal carinae nearly complete; laterope present; dorsope long and area laterally lamelliform; remainder of metasoma smooth and depressed; length of setose part of ovipositor sheath 0.12 times fore wing and 0.4 times as long as hind tibia and with a few long setae (Fig 1E); ovipositor sheath more or less widened subapically.

Colour. Dark orange; except following parts brown: ocellar triangle, apex of 3^rd^ antennal article, antennal articles 4^th^-18^th^ (and 19^th^-23^rd^ articles whitish) and metasoma (except first tergite); wing membrane slightly infuscate; pterostigma (but apically rather pale) and veins.

Variation. Antenna of ♀ with 20-23articles and 4-5 apical articles white and of ♂ with 18-19 articles; ovipositor sheath slightly widened subapically; fourth antennal article of ♀ 4.5–6.0 times as long as wide.

#### Distribution

South Korea.

#### Host

Collected in banana traps and in wild berries of *Rubus* infested by unidentified Drosophilidae.

#### Diagnostic remarks

The new species is similar to *A*. *orientalis* Viereck, 1913, reported from India, Korea and Philippines (Luzon) and to *A*. *pleuralis* (Ashmead) from Philippines, Indonesia, Japan and China. It differs by having apical 4-5 antennal articles white (apical 7-8 white in *A*. *orientalis*), ovipositor sheath more or less widened subapically (narrow subapically in *A*. *orientalis*),first metasomal tergite and mesoscutum dark orange (yellowish brown in *A*. *orientalis*); propodeum with rather short lamelliform costulae (without costulae in *A*. *orientalis*), third antennal segment of female about 4.5 times as long as wide (about 6.8 times in *A*. *orientalis*) and precoxal sulcus complete (absent anteriorly in *A*. *orientalis*). The length of ovipositor separates *A*. *brevicauda* from *A*. *pleuralis* as indicated in the key.

**Fig 2:**
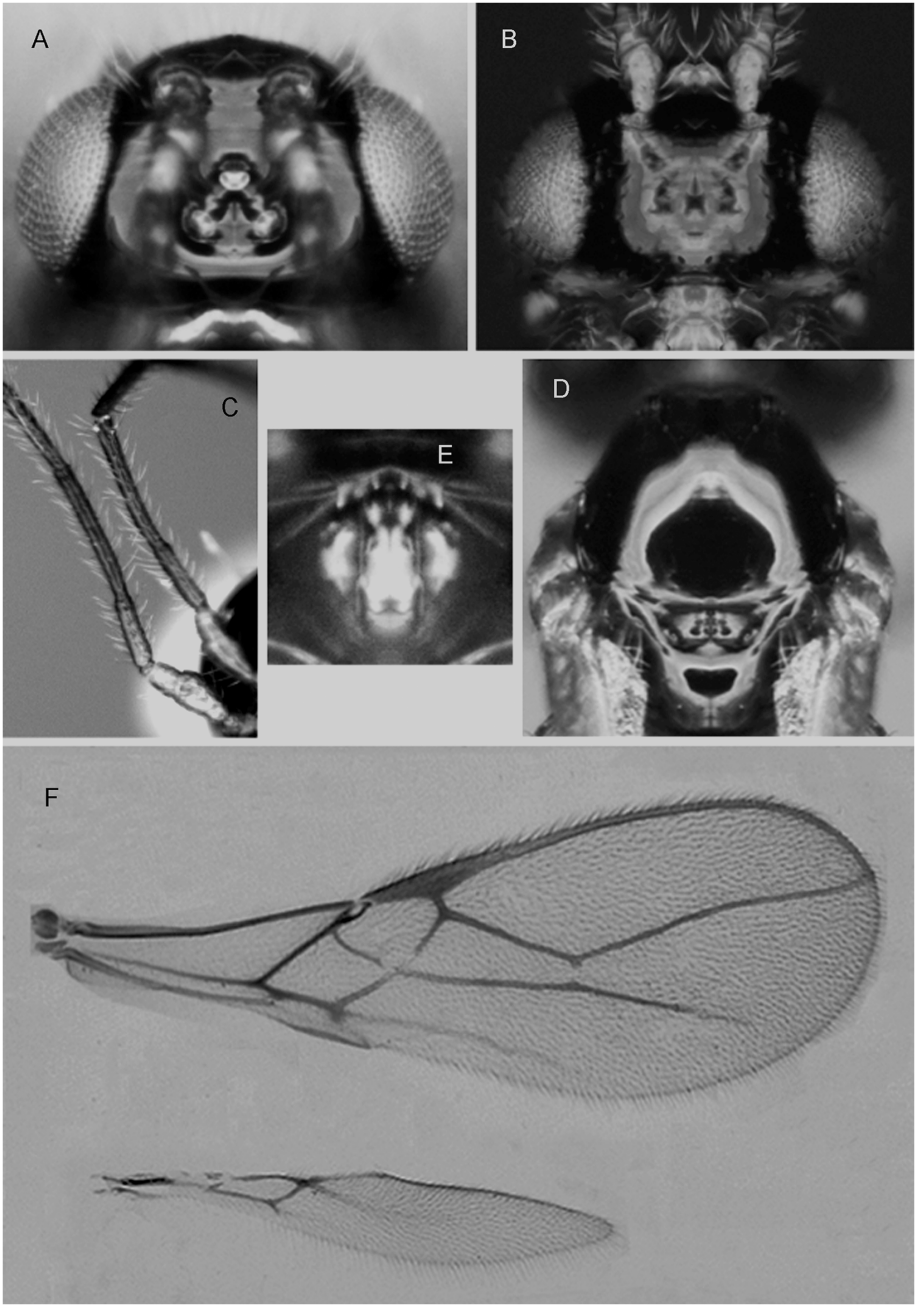
*Asobara elongata* sp. nov. ♀(A) Head in dorsal view. (B) Head in frontal view. (C) Basal segments of antenna. (D) Mesoscutum in dorsal view. (E) Areola.(F) Wings.

***Asobara elongata* van Achterberg and Guerrieri, sp. nov.** urn:lsid:zoobank.org:act:7B827FFC-4147-4997-AC57-D06E35BB3738 (Fig 2)

#### Material examined

**Type material**. Holotype, ♀, **China**, Yunnan, road to Kunming Zoological Experimental Station (25.10.17.32N 102.46.49.42E), 16.vii.2013, banana trap n. 9 (EGCZ6R BTR9-1, Dsz048 COI sequence KT835452) (Guerrieri, Giorgini, Wang). Holotype will be deposited at IZCAS.

Holotype, length of body (excluding ovipositor) 2.1 mm, length of fore wing 2.3 mm.

Head. Width of head 1.7 times its median length, largely glabrous and strongly shiny (Fig 2A and 2B); antenna incomplete (Fig 2C), densely erect bristly setose, length of third article 0.5 times as long as fourth article, slender, length of third and fourth articles 4.5 and 10.0 times their width, respectively; length of maxillary palp 1.2 times height of head; eye in dorsal view 3 times as long as temple, sparsely setose; temple in dorsal view gently curved (Fig 2A); OOL:diameter of ocellus: POL = 9:3:4 (Fig 2A); minimum width of face 0.54 times maximum width of head and 0.7 times its height, smooth, with some long and erect setae; clypeus transverse and rectangular, its surface smooth and flattened; height of clypeus in lateral view equal to width of middle tooth of mandible; anterior tentorial pits indistinct (Fig 2B); length of malar space 0.4 times basal width of mandible; mandible 2.5 times as long as wide, largely smooth, with a lamella ventrally and dorsally, with medium-sized carina connected to third tooth.

Mesosoma. Length of mesosoma 1.3 times its height; pronotum without pronope; antescutal depression distinct and finely crenulate laterally; side of pronotum and epicnemial area smooth; precoxal sulcus complete; remainder of mesopleuron smooth; episternal scrobe elliptical; pleural sulcus smooth, except for some crenulae medially and ventrally; mesosternal sulcus wide and with 3short crenulae; metapleuron largely smooth, except for a few carinae ventrally; notauli only anteriorly impressed, with strong lateral carina and largely absent on disc; medio-posterior depression of mesoscutum absent (Fig 2D); mesoscutum smooth and largely glabrous and its lateral carina almost complete, reaching tegulae; scutellar sulcus moderately deep and with one carina, 0.5 times as long as scutellum; scutellum in lateral view rather flat; metanotum slightly lamelliform protruding dorsally in lateral view; surface of propodeum smooth except for a medium-sized median carina, rather short lamelliform costulae and a parallel-sided areola (Fig 2E), its dorsal corners hardly protruding in lateral view and lateral carina of propodeum wide.

Wings. Pterostigma narrow triangular (Fig 2F); vein r 0.4 times width of pterostigma, issued from middle of pterostigma and vertical; r:3-SR:SR1 = 7:80:174; SR1 straight; cu-a obsolescent, postfurcal; 1-CU1:2-CU1 = 1:40; 2-SR:3-SR:r-m = 38:80:13; m-cu far antefurcal (2-SR+M 0.16 times as long as m-cu), forming an almost straight line with 2-SR+M, converging to 1-M. Hind wing: M+CU + 1-M:1r-m = 70:20; cu-a obsolescent.

Legs. Hind coxa smooth and elongate, rounded basally; tarsal claws slender medially; length of femur, tibia and basitarsus of hind leg 5.3, 11.0 and 3.5 times their width, respectively; hind femur, tibia and tarsus with long erect setae.

Metasoma. Length of first tergite twice its apical width, its surface largely smooth but coarsely rugose medially and laterally lamelliform, its dorsal carinae nearly complete; laterope present; dorsope long and area laterally lamelliform; remainder of metasoma smooth and depressed; setose part of ovipositor sheath0.42 times as long as fore wing and 1.1 times as long as hind tibia and with a few long setae; ovipositor sheath parallel-sided.

Colour. Body dark brown; following parts distinctly paler: clypeus, scapus, pedicellus, apical corners of mesopleuron and all legs; wing membrane slightly infuscate; pterostigma and veins pale brown.

Variation. Only one specimen available.**Distribution**. China (Yunnan).

#### Host

Collected in banana traps infested by unidentified Drosophilidae.

#### Diagnostic remarks

The new Oriental species is similar to the East Palaearctic *A*. *japonica*. It differs by having the fourth antennal article more elongate (about 10 times as long as wide, about 7 times in *A*. *japonica*), the propodeum black (yellowish brown or reddish) andthe head directly narrowed behind eyes (slightly less narrowed behind eye).

**Fig 3:**
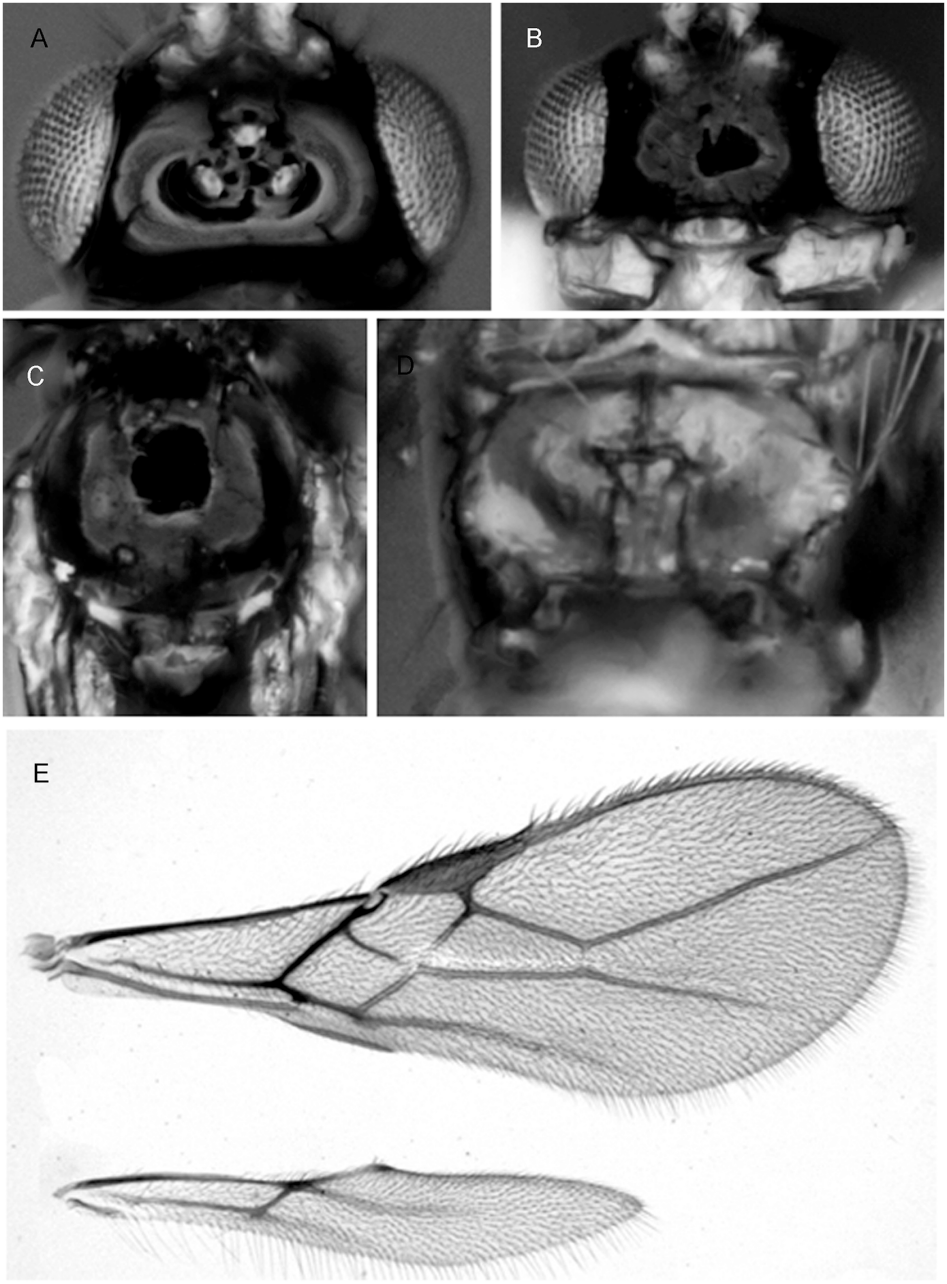
*Asobara japonica* Belokobylskij ♀(A) Head in dorsal view. (B) Head in frontal view. (C) Mesoscutum in dorsal view. (D) Areola. (E) Wings.

***Asobara japonica* Belokobylskij, 1998.** (Fig 3)

#### Material examined

11♀, 5♂, laboratory rearing on *Drosophila suzukii* from material collected in **SouthKorea**, Geochang, Gyeongsangnam-do (35°40'5.43"N 127°52'43.68"E), 11.viii.2014, ex Drosophilidae in melon/peach baited traps (Biondi); 6♀, South Korea, Boriamsa, Gyeongsangnam-do (35°40'5.43"N 127°52'43.68"E), 12.vii.2014, ex Drosophilidae in melon/peach bait (Dsz067 COI sequence KT835448, Dsz068 COI sequence KT835447; Dsz069 COI sequence KT835446, Dsz070 COI sequence KT835445, Dsz071 COI sequence KT835444 (Biondi); 14♀, South Korea, Boriamsa, Gyeongsangnam-do (35°40'5.43"N 127°52'43.68"E), 12.vii.2014, ex Drosophilidae in melon/peach baited traps (Biondi); 3♀, South Korea, Boriamsa, Gyeongsangnam-do (35°40'5.43"N 127°52'43.68"E), 12.vii.2014, ex Drosophilidae on wild *Rubus* (Dsz075 COI sequence KT835440; Dsz082 COI sequence KT835433)(Biondi); 1♀1♂ South Korea, Boriamsa, Gyeongsangnam-do (34° 45' 17.92'' N 127° 59' 29.38'' E), 12.vii.2014 ex Drosophilidae sp.on Melon/peach baited traps (Dsz080 COI sequence KT835435, Dsz081 COI sequence KT835434) (J.Miller, B.Miller, H. Reidl, Y.Song); 10♀, South Korea, Mangusan, Gyeongsangnam-do (34°50'56.38''N 127°51'13.76''E), 8.vii.2014, ex *Drosophila suzukii* on wild *Rubus* (Dsz073 COI sequence KT835442, Dsz074 COI sequence KT835441) (Biondi); 1♀, South Korea, Mangusan, Gyeongsangnam-do (34°50'56.38''N 127°51'13.76''E), 8.vii.2014, ex *Drosophila suzukii* on wild *Rubus*/sticky berries (Biondi); 1♀, South Korea, Gyeongsan, Gyeongsangnam-do (35°9'403056''N 128°5' 59.48''E), 10.vii.2014, ex *Drosophila suzukii* on wild *Rubus* (Biondi); 1♀, South Korea, Jagulsan, Gyeongsangnam-do (35°22'38.19''N 128°12'56.0''E), 10.vii.2014, ex *Drosophila suzukii* on wild *Rubus* (Biondi); 1♀, South Korea, Kumosan, Gyeongsangnam-do (35°0'33.65''N 127°51'57.94''E), 9.vii.2014, ex *Drosophila suzukii* on wild *Rubus*, (Dsz072 COI sequence KT835443)(Biondi); 2♀, South Korea, Geochang, Gyeongsangnam-do (35° 40' 5.43" N 127° 52' 43.68" E), 11.viii.2013 ex Drosophilidae from Melon/Banana baited traps and reared on *D*. *melanogaster* (Dsz078 COI sequence KT835437, Dsz079 COI sequence KT835436) (J.Miller, B.Miller); 1♀, South Korea, Mudeungsan Park entrance from the shopping street, road to monumental stones (35.08.346N 126.59.100E), 31.vii.2013, banana trap n. 3 (EGKUZ3 T31) (Guerrieri, Giorgini, Choi); 1♀, South Korea, Mudeungsan Park entrance from the shopping street, road to monumental stones (35.08.39.585N 126.59.13.308E), 31.vii.2013, banana trap n. 1 (EGKZ5BTR1-6, Dsz65)(Guerrieri, Giorgini, Choi); 2♀, South Korea, Geochang, South Gyeongsang (35° 40' 05", N 127° 52' 44"E), 15.viii.2013 ex *Drosophila melanogaster* from banana-fig-peach baited trap in blackberry farm (Dsz024 COI sequence KT835449) (J Miller, B Miller); 2♀, South Korea, Geochang, Gyeongsangnam-do (35° 40' 5.43" N 127° 52' 43.68" E), 11.viii.2013 ex Drosophilidae from Melon/Banana baited traps and reared on *D*. *suzuki* (Dsz076 COI sequence KT835439, Dsz077 COI sequence KT835438) (J Miller, B.Miller); 2♀, South Korea, Boriam, South Gyeongsang (34° 45' 23" N, 127° 58' 40" E), 15.viii.2013, ex *Drosophila melanogaster* from banana bait/ mixed-stand coastal forest (Dsz026)(J Miller, B Miller); 2♀ South Korea, Busan, Seo-gu Mt.Gudeoksan (35.07.32N 129.00.31E), Apple trap,1.vii.2013 (Dsz017 COI sequence KT835451, Dsz018 COI sequence KT835450)(D. Choi).

#### Redescription

Length of body (excluding ovipositor) 1.8 mm, length of fore wing 2.1 mm.

Head. Width of head 1.5 times its median length, largely glabrous and shiny; antenna with 21 articles and densely erect bristly setose, length of third article 0.6 times as long as fourth article, slender, length of third, fourth and penultimate articles 4.5, 7.0 and 3.0 times their width, respectively; length of maxillary palp 1.4 times height of head; eye in dorsal view 4 times as long as temple, sparsely setose; temple in dorsal view subparallel-sided (Fig 3A); OOL:diameter of ocellus:POL = 7:3:4 (Fig 3A); minimum width of face 0.52 times maximum width of head and 1.2 times its height (Fig 3B), evenly smooth, with some long and erect setae; clypeus transverse, oval, its surface smooth and flattened; height of clypeus in lateral view equal to width of middle tooth of mandible; anterior tentorial pits indistinct (Fig 3B); length of malar space 0.1 times basal width of mandible; mandible 1.9 times as long as wide, largely smooth, with a lamella ventrally and dorsally, medium-sized carina connected to third tooth.

Mesosoma. Length of mesosoma 1.1 times its height; pronotum without pronope; antescutal depression distinct and finely crenulate laterally; side of pronotum smooth; epicnemial area smooth; precoxal sulcus complete; remainder of mesopleuron smooth; episternal scrobe elliptical; pleural sulcus smooth, except for some crenulae medially and ventrally; mesosternal sulcus wide and with 1 short crenula; metapleuron largely smooth, except for a few carinae ventrally; notauli (Fig 3C) only anteriorly impressed, with strong lateral carina and largely absent on disc; mesoscutum smooth and largely glabrous and its lateral carina almost complete, reaching tegulae; scutellar sulcus moderately deep and with one carina, as long as scutellum; scutellum in lateral view rather flat; metanotum slightly lamelliform protruding dorsally in lateral view; surface of propodeum smooth except for a medium-sized median carina, rather short lamelliform costulae and a wide parallel shaped areola (Fig 3D), its dorsal corners hardly protruding in lateral view and lateral carina of propodeum wide; propodeal spiracle round, small and in front of apical third of propodeum.

Wings. Pterostigma narrow triangular (Fig 3E); vein r 0.4 times as width of pterostigma, issued in the middle of pterostigma and vertical; r:3-SR:SR1 = 9:59:165; SR1 straight; cu-a obsolescent, postfurcal; 1-CU1:2-CU1 = 4:6; 2-SR:3-SR:r-m = 40:59:11; m-cu merging with 2-SR, converging to 1-M, 2-SR+M obsolescent. Hind wing: M+CU + 1-M:1r-m = 90:20; cu-a present.

Legs. Hind coxa smooth and elongate, rounded basally; tarsal claws slender medially; length of femur, tibia and basitarsus of hind leg 5.5, 11 and 3 times their width, respectively; hind femur, tibia and tarsus with long erect setae.

Metasoma. Length of first tergite 2.25 times its apical width, its surface largely smooth but coarsely rugose medially and laterally lamelliform, its dorsal carinae nearly complete; laterope present; dorsope long and area laterally lamelliform; remainder of metasoma smooth and depressed; length of setose part of ovipositor sheath 0.4 times fore wing and 1.1 times as long as hind tibia and with a few long setae; apex of ovipositor sheath parallel-sided medially.

Colour. Body brown except: yellowish clypeus and inter-torular area; scapus and pedicellus yellow (17^th^antennal article grey, 18^th^-21^st^articles white), propleuron and upper corner of mesopleuron, first metasomal tergite; mesoscutum, scutellum and propodeum suffused with yellow; wing membrane slightly infuscate; pterostigma brown (but rather pale at base and apex), veins mostly brown, legs yellow, ovipositor sheath brown and apically whitish.

Variation. Not much in the material at hand apart from: antenna of ♂ with 2-3 and of ♀ with 4-5 white apical articles; white apical articles of antenna of ♀ with some more or less dark setae apically; setose part of ovipositor sheath 0.45-0.50 times as long as fore wing and 1.1-1.2 times as long as hind tibia; pterostigma brown to dark brown medially; wing membrane rather infuscate; mesoscutum and metapleuron of ♀ with brown parts appearing black in darker specimens.

#### Host

Collected in banana traps infested by *D*. *suzukii* and/or by unidentified Drosophilidae

#### Distribution

*South Korea, Russia (Far East), Japan.

**Fig 4:**
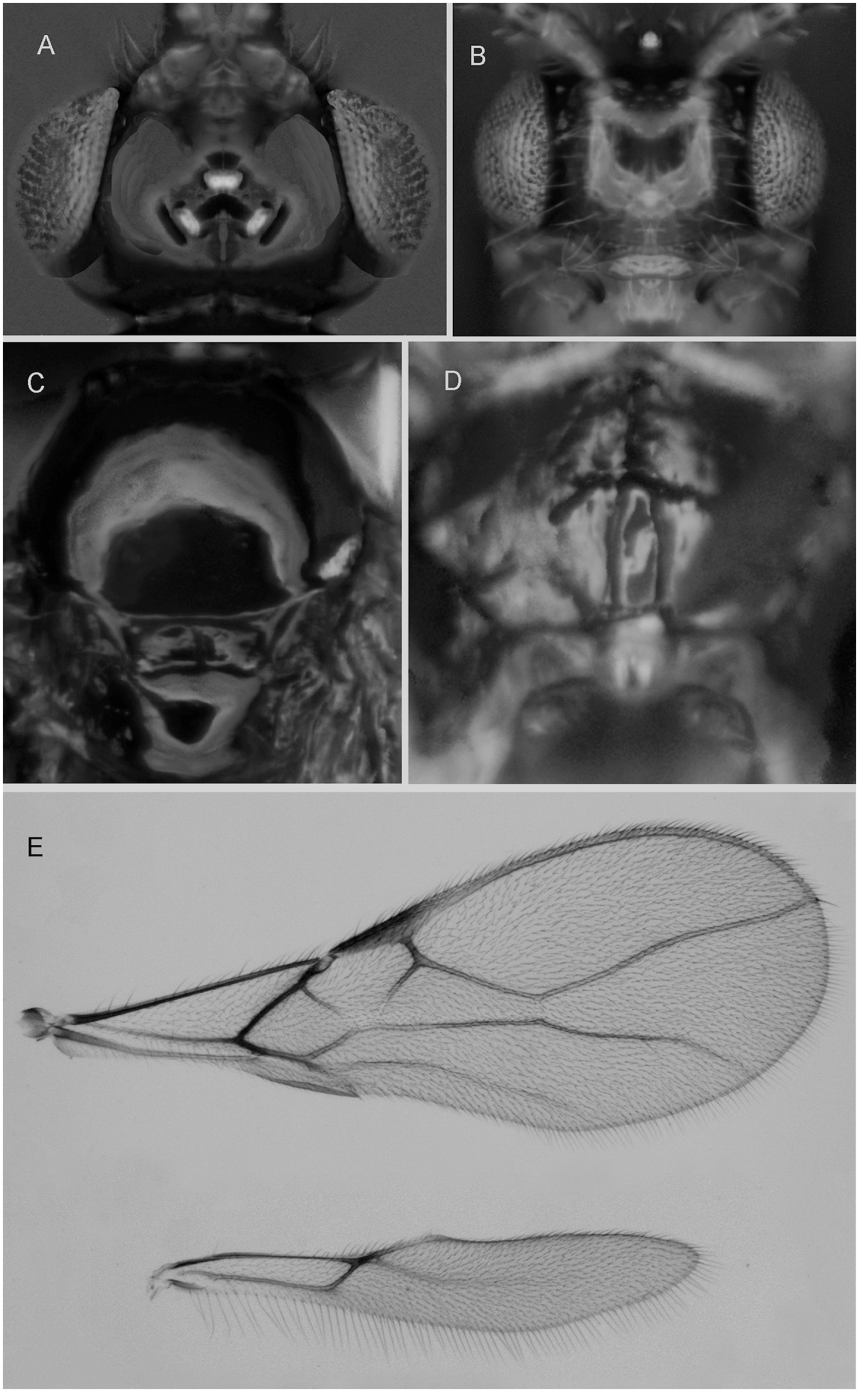
*Asobara mesocauda* sp. nov. ♀ (A) Head in dorsal view. (B) Head in frontal view. (C) Mesoscutum in dorsal view. (D) Areola. (E) Wings.

***Asobara mesocauda* van Achterberg and Guerrieri, sp. nov.** urn:lsid:zoobank.org:act:B52F38C6-EBD9-439A-A766-C12E9D0E6DA5 (Fig 4)

#### Material examined

**Type material**. Holotype, ♀, **China**, Yunnan, road to Kunming Zoological Experimental Station (25.10.35.53N 102.47.40.87E), 16.vii.2013, banana trap n. 13 (EGCZ6R BTR13-4 COI sequence Dsz014-KT835422) (Guerrieri, Giorgini, Wang). Paratypes: 2♀, China, Yunnan, road to Kunming Zoological Experimental Station (25.10.35.53N 102.47.40.87E), 16.vii.2013, banana trap n. 13 (EGCZ6R BTR13-9) (Guerrieri, Giorgini, Wang); 1♀, China, Yunnan, road to Kunming Zoological Experimental Station (25.11.18.82N 102.48.13.33E), 16.vii.2013, banana trap n. 18 (EGCZ6R BTR18-2, Dsz056 COI sequence KT835418) (Guerrieri, Giorgini, Wang); 1♀,id., but banana trap n. 19 (EGCZ6R BTR19-3); 1♀,id., but banana trap n. 20 (EGCZ6R BTR20-2); 1♀, China, Yunnan, Xi Shian Forest (Sleeping Princess) (24.58.27.08N 102.37.30.82E), 18.vii.2013, banana trap n. 4, (EGCZ7 BTR4-9)(Guerrieri, Giorgini, Wang); 1♀,id., but banana trap n. 5, (EGCZ7 BTR5-2); 1♀,id., but banana trap n. 11, (EGCZ7 BTR11-6, Dsz012 COI sequence KT835424).

**Non type material:** 3♀, China, Yunnan, road to Kunming Zoological Experimental Station (25.10.35.53N 102.47.40.87E), 16.vii.2013, banana trap n. 13 (EGCZ6R BTR13-8 + BTR13-11, Dsz052 COI sequence KT835419) (Guerrieri, Giorgini, Wang); 1♀, China, Yunnan, road to Kunming Zoological Experimental Station (25.10.55.07N 102.47.52.51E), 16.vii.2013, banana trap n. 15 (EGCZ6R BTR15-2) (Guerrieri, Giorgini, Wang); 1♀, China, Yunnan, road to Kunming Zoological Experimental Station (25.10.44.22N 102.47.19.44E), 16.vii.2013, banana trap n. 12 (EGCZ6R BTR12-4) (Guerrieri, Giorgini, Wang); 2♀, China, Yunnan, road to Kunming Zoological Experimental Station (25.10.17.32N 102.46.49.42E), 16.vii.2013, banana trap n. 9 (EGCZ6R BTR9-7, Dsz013 COI sequence KT835423; EGCZ6R BTR9-10, Dsz049 COI sequence KT835421) (Guerrieri, Giorgini, Wang); 1♀, China, Yunnan, road to Kunming Zoological Experimental Station (25.11.32.61N 102.49.47.92E), 16.vii.2013, banana trap n. 13 (EGCZ6R BTR13-6, Dsz051 COI sequence KT835420) (Guerrieri, Giorgini, Wang); 3♀, China, Yunnan, Xi Shian Forest (Sleeping Princess) (24.58.27.08N 102.37.30.82E), 18.vii.2013, banana trap n. 4, (EGCZ7 BTR4-2,3,5 Dsz010 COI sequence KT835426)(Guerrieri, Giorgini, Wang); 4♀, China, Yunnan, Xi Shian Forest (Sleeping Princess) (24.58.26.07N 102.37.37.20E), 18.vii.2013, banana trap n. 5, (EGCZ7 BTR5-5, Dsz061 COI sequence KT835414; EGCZ7 BTR5-1 Dsz060 COI sequence KT835415, EGCZ7 BTR5-4, EGCZ7 BTR5-6)(Guerrieri, Giorgini, Choi); 1♀, China, Kunming Botanical Gardens, zone fruit/ornamental plants (25.08.42.27N 102.44.30.72E), 15.vii.2013, banana trap n. 3, (EGCZ5 BTR3-2, Dsz059 COI sequence KT835416) (Guerrieri, Giorgini, Wang);1♀, China, Yunnan, Xi Shian Forest (Sleeping Princess) (24.58.20.11N 102.37.40.77E), 18.vii.2013, banana trap n. 6, (EGCZ7 BTR6-1)(Guerrieri, Giorgini, Wang); 2♀, China, Yunnan, Xi Shian Forest (Sleeping Princess) (24.57.53.86N 102.37.44.83E), 18.vii.2013, banana trap n. 11, (EGCZ7 BTR11-1, Dsz011 COI sequence KT835425; EGCZ7 BTR11-4)(Guerrieri, Giorgini, Wang); 1♀, China, Kunming Botanical Gardens, zone fruit/ornamental plants (25.08.20.41N 102.44.26.46E), 15.vii.2013, banana trap n. 3, (EGCZ5 BTR3-1, Dsz058 COI sequence KT835417)(Guerrieri, Giorgini, Wang); 1♀, China, Kunming Botanical Gardens, zone fruit/ornamental plants (25.08.42.27N 102.44.30.72E), 15.vii.2013, banana trap n. 7, (EGCZ5 BTR7-1)(Guerrieri, Giorgini, Wang); 1♀, China, Shi Lin County Kunming City Da Mogu Town Resort Kang Yi Zu, blueberry field(25.08.42.27N 102.44.30.72E), 13.vii.2013, banana trap n. 11, (EGCZ4 BTR11)(Guerrieri, Giorgini, Wang). Holotype will be deposited at IZCAS, paratypes will be deposited at RMNH.

Holotype, length of body (excluding ovipositor) 1.9 mm, length of fore wing 2.1 mm.

Head. Width of head 1.4 times its median length, largely glabrous and strongly shiny (Fig 4A and 4B); antenna with 21 articles, densely erect bristly setose, length of third article 0.6 times as long as fourth article, slender, length of third, fourth and penultimate articles 3.5 5.5 and 3.0 times their width, respectively; length of maxillary palp 1.1 times height of head; eye in dorsal view 3 times as long as temple, sparsely setose; temple in dorsal view gently curved (Fig 4A); OOL:diameter of ocellus:POL = 7:2:3 (Fig 4A); minimum width of face 0.56 times maximum width of head and 0.8 times its height, smooth, with some long and erect setae; clypeus transverse and oval, its surface smooth and flattened (Fig 4B); height of clypeus in lateral view equal to width of middle tooth of mandible; anterior tentorial pits indistinct (Fig 4B); length of malar space 0.1 times basal width of mandible; mandible 1.9 times as long as wide, largely smooth, with a lamella ventrally and dorsally, with medium-sized carina connected to third tooth.

Mesosoma. Length of mesosoma 1.2 times its height; pronotum without pronope; antescutal depression distinct and finely crenulate laterally; side of pronotum smooth; epicnemial area smooth; precoxal sulcus complete; remainder of mesopleuron smooth; episternal scrobe elliptical; pleural sulcus smooth, except for some crenulae medially and ventrally; mesosternal sulcus wide and with 1 short crenula; metapleuron largely smooth, except for a few carinae ventrally; notauli only anteriorly impressed, with strong lateral carina and largely absent on disc; medio-posterior depression of mesoscutum absent (Fig 4C); mesoscutum smooth and largely glabrous and its lateral carina almost complete, reaching tegulae; scutellar sulcus moderately deep, 0.5 times as long as scutellum and with one carina; scutellum in lateral view rather flat; metanotum slightly lamelliform protruding dorsally in lateral view; surface of propodeum smooth except for a medium-sized median carina, rather short lamelliform costulae and a narrow parallel-sided areola (Fig 4D), its dorsal corners hardly protruding in lateral view and lateral carina of propodeum wide.

Wings. Pterostigma narrow triangular (Fig 4E); vein r 0.8 times width of pterostigma, issued from basal 0.3 of pterostigma and vertical; r:3-SR:SR1 = 13:68:162; SR1 straight; cu-a obsolescent, postfurcal; 1-CU1:2-CU1 = 8:25; 2-SR:3-SR:r-m = 40:68:10; m-cu far antefurcal (2-SR+M 0.3 times as long as m-cu), forming an almost straight line with 2-SR+M, converging to 1-M. Hind wing: M+CU + 1-M:1r-m = 87:18; cu-a absent.

Legs. Hind coxa smooth and elongate, rounded basally; tarsal claws slender medially; length of femur, tibia and basitarsus of hind leg 6.4, 10.6 and 2.8 times their width, respectively; hind femur, tibia and tarsus with long erect setae.

Metasoma. Length of first tergite 1.6 times its apical width, its surface largely smooth but coarsely rugose medially and laterally lamelliform, its dorsal carinae nearly complete; laterope present; dorsope long and area laterally lamelliform; remainder of metasoma smooth and depressed; length of setose part of ovipositor sheath 0.34 times fore wing and about as long as hind tibia and with a few long setae; ovipositor sheath parallel-sided.

Colour. Body dark brown; with following parts paler: face and clypeus, scapus (and 18^th^-21^st^ articles whitish), apical corner of mesopleuron and all legs; wing membrane slightly infuscate; pterostigma and veins brown.

Variation. Antenna of ♀ with 3-4 white apical articles. Face sometimes bright yellow, apical part of mesopleuron sometimes yellow (heavily contrasting with the remaining dark brown part)

#### Distribution

China (Yunnan).

#### Host

Collected in banana traps infested by unidentified Drosophilidae

#### Diagnostic remarks

The new Oriental species is similar to the East Palaearctic *A*. *japonica* Belokobylskij. It differs by having the face largely or completely brownish yellow, similar to colour of the clypeus (face dark brown, strongly contrasting with yellowish clypeus in *A*. *japonica*), first discal cell of the fore wing slightly more elongate (rather robust) and setose part of ovipositor sheath about as long as hind tibia (1.1–1.2 times as long as hind tibia).

**Fig 5:**
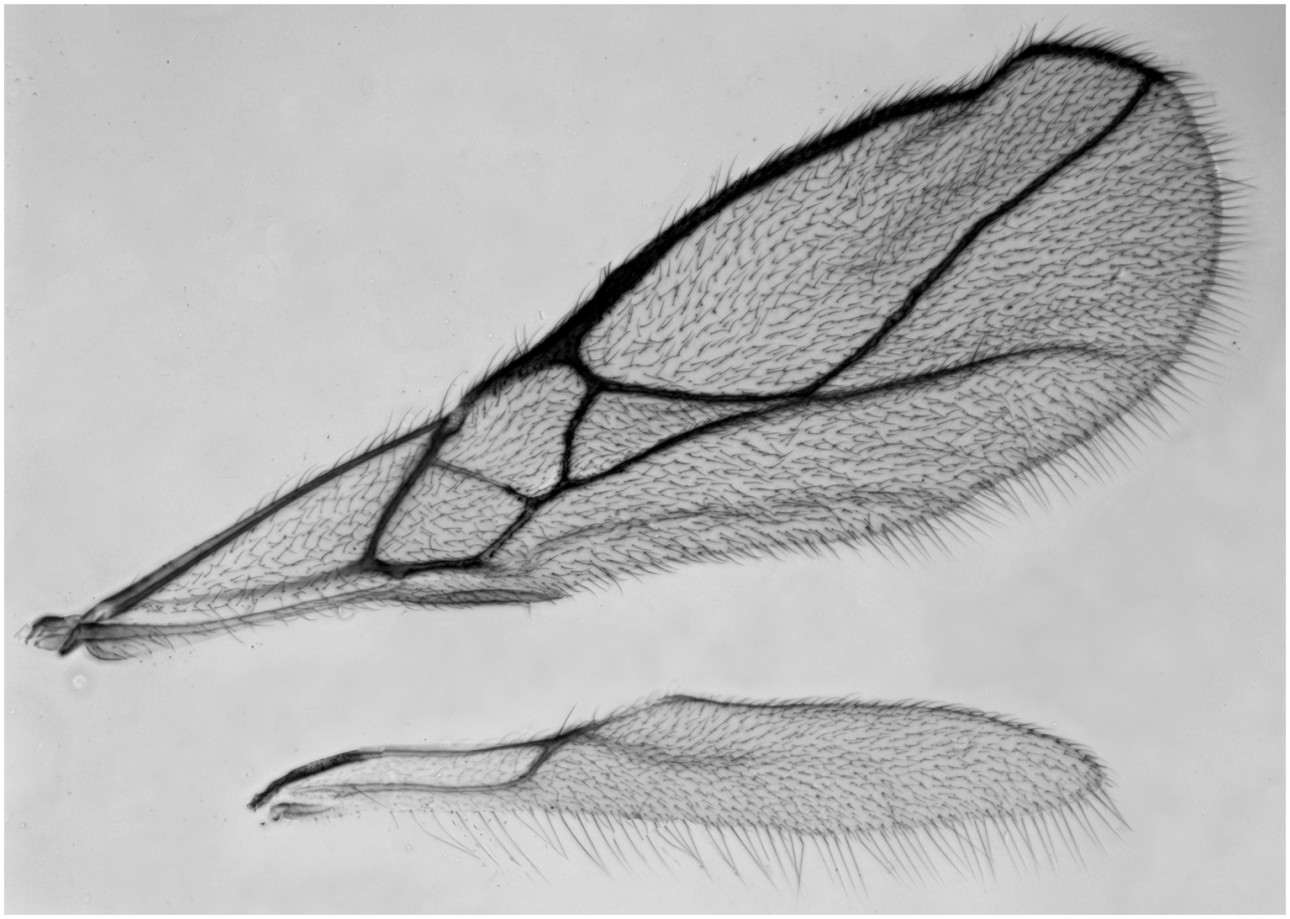
*Asobara triangulata* sp. nov.♀. Wings.

***Asobara triangulata* van Achterberg and Guerrieri, sp. nov.** urn:lsid:zoobank.org:act:0E7023F4-1F9B-4702-B45D-9E2C224FAE8D (Fig 5)

#### Material examined

**Type material**. Holotype, ♀, **China**, Yunnan, Xi Shian Forest (Sleeping Princess) (24.58.26.07N 102.37.37.20E), 18.vii.2013, banana trap n. 5, (EGCZ7 BTR5-7-Dsz062 COI sequence KT835413) (Guerrieri, Giorgini, Wang). Holotype will be deposited at IZCAS.

Holotype, length of body (excluding ovipositor) 1.9 mm, length of fore wing 2.1 mm.

Head. Width of head 1.4 times its median length, largely glabrous and strongly shiny; antenna with 19 articles, densely erect bristly setose, length of third article 0.6 times as long as fourth article, slender, length of third, fourth and penultimate articles 3.5 5.5 and 3.0 times their width, respectively; length of maxillary palp 1.1 times height of head; eye in dorsal view 3 times as long as temple, sparsely setose; temple in dorsal view gently curved; OOL:diameter of ocellus:POL = 7:2:3; minimum width of face 0.56 times maximum width of head and 0.8 times its height, smooth, with some long and erect setae; clypeus transverse and oval, its surface smooth and flattened; height of clypeus in lateral view equal to width of middle tooth of mandible; anterior tentorial pits indistinct; length of malar space 0.1 times basal width of mandible; mandible 1.9 times as long as wide, largely smooth, with a lamella ventrally and dorsally, with medium-sized carina connected to third tooth.

Mesosoma. Length of mesosoma 1.2 times its height; pronotum without pronope; antescutal depression distinct and finely crenulate laterally; side of pronotum smooth; epicnemial area smooth; precoxal sulcus complete; remainder of mesopleuron smooth; episternal scrobe elliptical; pleural sulcus smooth, except for some crenulae medially and ventrally; mesosternal sulcus wide and with 1 short crenula; metapleuron largely smooth, except for a few carinae ventrally; notauli only anteriorly impressed, with strong lateral carina and largely absent on disc; medio-posterior depression of mesoscutum absent; mesoscutum smooth and largely glabrous and its lateral carina almost complete, reaching tegulae; scutellar sulcus moderately deep, 0.5 times as long as scutellum and with one carina; scutellum in lateral view rather flat; metanotum slightly lamelliform protruding dorsally in lateral view; surface of propodeum smooth except for a medium-sized median carina, rather short lamelliform costulae and a narrow parallel-sided areola, its dorsal corners hardly protruding in lateral view and lateral carina of propodeum wide.

Wings. Pterostigma narrow triangular (Fig 5); vein r 0.8 times width of pterostigma, issued from basal 0.3 of pterostigma and vertical; r:3-SR:SR1 = 9:57:1130; SR1 straight; cu-a obsolescent, postfurcal; 1-CU1:2-CU1 = 6:20; 2-SR:3-SR:r-m = 29:57:2; m-cu far antefurcal (2-SR+M 0.45 times as long as m-cu), forming an almost straight line with 2-SR+M, converging to 1-M. Hind wing: M+CU + 1-M:1r-m = 65:10; cu-a absent.

Legs. Hind coxa smooth and elongate, rounded basally; tarsal claws slender medially; length of femur, tibia and basitarsus of hind leg 6.4, 10.6 and 2.8 times their width, respectively; hind femur, tibia and tarsus with long erect setae.

Metasoma. Length of first tergite 1.6 times its apical width, its surface largely smooth but coarsely rugose medially and laterally lamelliform, its dorsal carinae nearly complete; laterope present; dorsope long and area laterally lamelliform; remainder of metasoma smooth and depressed; length of setose part of ovipositor sheath 0.34 times fore wing and about as long as hind tibia and with a few long setae; ovipositor sheath parallel-sided.

Colour. Body dark brown; with following parts paler: face and clypeus, scapus (and 15^th^-17^th^articles whitish), apical corner of mesopleuron and all legs; wing membrane slightly infuscate; pterostigma and veins brown.

Variation. Only one specimen available.

#### Distribution

China (Yunnan), Japan

#### Host

Collected in banana traps infested by unidentified Drosophilidae

#### Diagnostic remarks

The single specimen examined appears almost identical to *A*. *mesocauda* if not for the shorter r-m vein in the fore wing and for antenna of 19 segments (21 in *A*. *mesocauda*). These differences appear to be supported by molecular evidence (see below) even though it is possible that more morphological differences will emerge when more material will be available.

**Fig 6:**
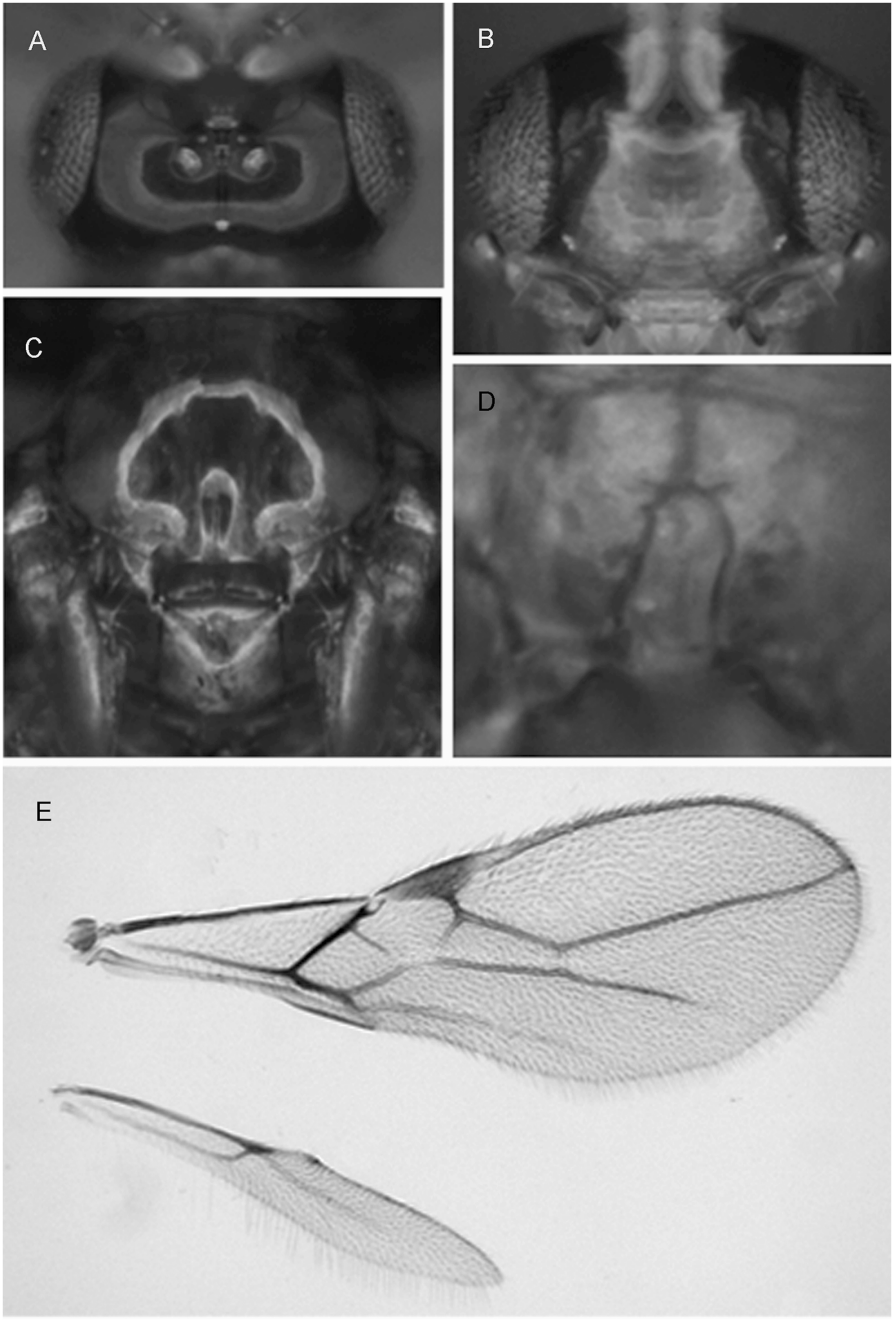
*Asobara leveri* (Nixon) ♀ (A) Head in dorsal view. (B) Head in frontal view. (C) Mesoscutum in dorsal view. (D) Areola. (E) Wings.

***Asobara leveri* (Nixon, 1939).** (Fig 6)

#### Material examined

3♀, **SouthKorea**, Manhaengsan (little artificial lake with temple) (35.32.02.456N 127.26.13.929E), 29.vii.2013, banana trap n. 17 (EGKUZ3 T171,172,174) (Guerrieri, Giorgini, Choi); 1♀, South Korea, Mudeungsan Park entrance from the shopping street, road to monumental stones (35.08.158N 126.59.156E), 31.vii.2013, banana trap n. 7 (EGKUZ3 T71) (Guerrieri, Giorgini, Choi); 1♀, South Korea, Mudeungsan Park entrance from the shopping street, road to monumental stones (35.08.518N 126.59.034E), 31.vii.2013, banana trap n. 2 (EGKZ5BTR2-2, Dsz016 COI sequence KT835432) (Guerrieri, Giorgini, Choi); 2♀, South Korea, Uigoksa, South Gyeongsang (35°12'15"N 128°04'54"E, 17.viii.2013, fruit bait (PW Shearer, JC Miller, B Miller); 1♀, South Korea, Namjangsa, North Gyeongsang (36°25'42"N 128°06'26"E), 15.viii.2013, fruit baited trap with *D*. *suzukii* (EGCK5 Dsz022 COI sequence KT835429) (JC Miller, B Miller); 2♀, South Korea Boriam South Gyeongsang (34° 45' 23"N, 127° 58' 40" E), 15.viii.2013, Banana baited trap infested with *D*. *melanogaster* mixed-stand coastal forest (EGCK1 Dsz020 COI sequence KT835431; EGCK3—Dsz21 COI sequence KT835430) (J Miller, B Miller); 1♀, South Korea, Namjangsa North Gyeongsang (36° 25' 42"N, 128° 06' 26"E), 15.viii.2013, melon-banana-blueberry baited trap infested with *D*. *melanogaster*, mixed-stand inland forest (EGCK6—Dsz023 COI sequence KT835428) (J Miller, B Miller); 1♀, South Korea, Boriamsa Gyeongsangnam-do (34° 45' 17.92'' N 127° 59' 29.38'' E), 12.VII.2014 Melon/peach baited trap infested by Drosophilidae sp. (Dsz084 COI sequence KT835427) (J Miller, B Miller, H Reidl, Y Song).

#### Redescription

Length of body (excluding ovipositor) 1.6 mm, length of fore wing 1.8 mm.

Head. Width of head 1.3 times its median length, largely glabrous and strongly shiny (Fig 6A and 6B); antenna with 20 articles, densely erect bristly setose, length of third article 0.7 times as long as fourth article, slender, length of third, fourth and penultimate articles 4.0, 6.0 and 3.5 times their width, respectively; length of maxillary palp 1.1 times height of head; eye in dorsal view 3.5 times as long as temple, sparsely setose; temple in dorsal view subparallel-sided (Fig 6A); OOL:diameter of ocellus:POL = 6:2:3 (Fig 6A); minimum width of face 0.55 times maximum width of head and 0.85 times its height, smooth, with some long and erect setae; clypeus transverse, sub-elliptical, its surface smooth and flattened; height of clypeus in lateral view equal to width of middle tooth of mandible; anterior tentorial pits indistinct (Fig 6B); length of malar space 0.1 times basal width of mandible; mandible 1.9 times as long as wide, largely smooth, with a lamella ventrally and dorsally, with medium-sized carina connected to third tooth.

Mesosoma. Length of mesosoma 1.3 times its height; pronotum without pronope; antescutal depression distinct and finely crenulate laterally; side of pronotum smooth; epicnemial area smooth; precoxal sulcus complete; remainder of mesopleuron smooth; episternal scrobe elliptical; pleural sulcus smooth, except for some crenulae medially and ventrally; mesosternal sulcus wide and with 3 short crenulae; metapleuron largely smooth, except for a few carinae ventrally; notauli only anteriorly impressed, with strong lateral carina and largely absent on disc; medio-posterior depression of mesoscutum elongated, deep and elliptical (Fig 6C) and lateral carina absent in front of tegulae; mesoscutum smooth and largely glabrous and its lateral carina almost complete, reaching tegulae; scutellar sulcus moderately deep, as long as scutellum and with one carina; scutellum in lateral view rather flat; metanotum slightly lamelliform protruding dorsally in lateral view; surface of propodeum smooth except for a medium-sized median carina, rather short lamelliform costulae and a wide parallel-sided areola (Fig 6D), its dorsal corners hardly protruding in lateral view and lateral carina of propodeum wide.

Wings. Pterostigma narrow triangular (Fig 6E); vein r 0.6 times width of pterostigma, issued from basal 0.3 of pterostigma and vertical; r:3-SR:SR1 = 9:55:155; SR1 straight; cu-a obsolescent, postfurcal; 1-CU1:2-CU1 = 2:5; 2-SR:3-SR:r-m = 23:55:10; m-cu far antefurcal (2-SR+M 0.33 times as long as m-cu), forming an almost straight line with 2-SR+M, converging to 1-M. Hind wing: M+CU + 1-M:1r-m = 85:20; cu-a absent.

Legs. Hind coxa smooth and elongate, rounded basally; tarsal claws slender medially; length of femur, tibia and basitarsus of hind leg 4.8,10.7 and 3.0 times their width, respectively; hind femur, tibia and tarsus with long erect setae.

Metasoma. Length of first tergite 1.9 times its apical width, its surface largely smooth but coarsely rugose medially and laterally lamelliform, its dorsal carinae nearly complete; laterope present; dorsope long and area laterally lamelliform; remainder of metasoma smooth and depressed; length of setose part of ovipositor sheath 0.38 times fore wing and slightly longer than hind tibia and with a few long setae; ovipositor sheath parallel-sided.

Colour. Head brown with face and clypeus yellow, stemmaticum (ocellar triangle) appearing darker; 1^st^-3^rd^ antennal articles yellow, 17^th^-20^th^ articles whitish, remaining articles dark brown, mesosoma yellow brown (paler than head) with darker areas in front of mesoscutum, basal half of mesopleuron dark brown and remaining part yellow brown; propodeum and metasoma brown but first tergite appearing paler; wing membrane slightly infuscate; pterostigma light brown (but rather pale at base and apex), veins mostly pale brown, legs yellow, ovipositor sheath brown.

Variation. Not much in the few specimens at hand.

#### Host

Collected in fruit traps infested by *D*. *suzukii*, *D*. *melanogaster* and unidentified Drosophilidae.

#### Distribution

*South Korea, China (Hubei), Fiji, Japan

#### *Asobara pleuralis* (Ashmead)

**Material examined:** 10♀ **Philippines**, ex Drosophila sp., no. 308., in lab., M. v.d. Linden, RMNH'03"; 5♀, Philippines, Isabela, Bintacan, cult. in lab. from *Drosophila ananassae* Dol., em. v.1995, J. Ellers, RMNH'95"; 1♀, Philippines, Luzon, Ilagan, Bintacan, Bintacan River, c 1 km NE of Binta, autumn 1992, ex *Drosophila* sp. in fermenting fruit, M. v.d. Linden, RMNH'93; 5♀, **Indonesia**, Java, Bogor, c. 400 m, cult. in lab. from *Drosophila ananassae* Dol., em. v. 1995, J. Ellers, RMNH"; 5♀ **Thailand**, N. Thailand, Mae Hong Son, c 550 m, cult. in lab. from *Drosophila ananassae* Dol., em. v.1995, J. Ellers, RMNH'95"; 2♀, S. Thailand, Bangkok, cult. in lab. from *Drosophila ananassae* Dol., em. v.1995, J. Ellers, RMNH'95.

**Diagnostic remarks:** The species has been reliably described and pictured by Fischer [26].

**Fig 7:**
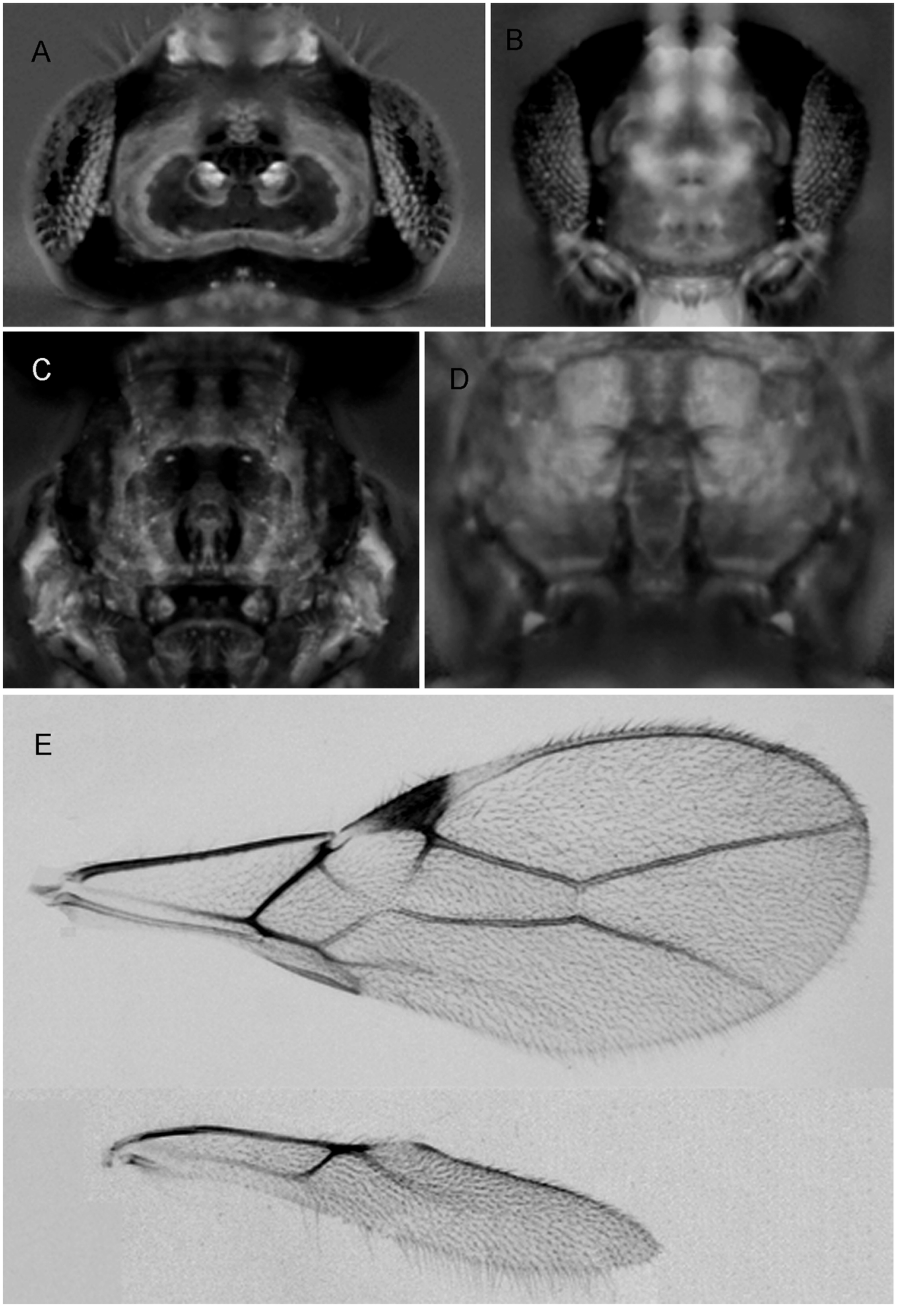
*Asobara unicolorata* sp. nov. ♀ (A) Head in dorsal view. (B) Head in frontal view. (C) Mesoscutum in dorsal view. (D) Areola. (E) Wings.

***Asobara unicolorata* van Achterberg and Guerrieri, sp. nov.** urn:lsid:zoobank.org:act:4B290523-7E37-456B-9B33-8AA472CE7E18 (Fig 7)

#### Type material

Holotype, ♀, laboratory rearing from material collected in **South Korea**, Manhaengsan (little artificial lake with temple) (35.32.02.456N 127.26.13.929E), 29.vii.2013, banana trap n. 17 (EGKUZ3T172) (Guerrieri, Giorgini, Choi). Paratypes: 1♀, South Korea, Manhaengsan (35.08.268N 126.59.088E), 31.vii.2015, banana trap n. 5 (EGKZ5BTR5-2) (Guerrieri, Giorgini, Choi); 1♀, **China**, Yunnan, road to Kunming Zoological Experimental Station (25.11.23.78N 102.48.17.42E), 16.vii.2013, banana trap n. 19 (EGCZ6R BTR19-1-Dsz055 COI sequence KT835410) (Guerrieri, Giorgini, Choi); 1♀, China, Yunnan, Chengjian County Yuxi City (24.42.42.89N 102.52.15.11E), 12.vii.2013, banana trap n. 1, (EGCZ1 BTRB-1 Dsz006 COI sequence KT835412) (Guerrieri, Giorgini, Wang); 1♀, China, Yunnan, Chengjian County Yuxi City (24.42.46.67N 102.52.05.39N), 12.vii.2013, banana trap n. 1, (EGCZ1 BTR7-1 Dsz083 COI sequence KT835409) (Guerrieri, Giorgini, Wang), 1♀, China, Yunnan, Xi Shian Forest (Sleeping Princess) (24.58.20.11N 102.37.40.77E), 18.vii.2013, banana trap n. 6, (EGCZ7 BTR6-3 Dsz053 COI sequence KT835411) (Guerrieri, Giorgini, Wang). Holotype and 1 paratype will be deposited at RMNH, 3 paratypes will be deposited at IZCAS.

Holotype, length of body (excluding ovipositor) 1.9 mm, length of fore wing 2.1 mm.

Head. Width of head 1.4 times its median length, largely glabrous and strongly shiny dorsally (Fig 7A); antenna with 21 articles, densely erect bristly setose, length of third article 0.7 times as long as fourth article, slender, length of third, fourth and penultimate articles 4.5 6.5 and 4.0 times their width, respectively; length of maxillary palp 1.3 times height of head; eye in dorsal view 2.8 times as long as temple, sparsely setose; temple in dorsal view subparallel-sided; OOL:diameter of ocellus:POL = 7:3:3 (Fig 7a); minimum width of face 0.5 times maximum width of head and 0.7 times its height, smooth, with some long and erect setae; clypeus transverse and oval, its surface smooth and flattened (Fig 7b); height of clypeus in lateral view equal to width of middle tooth of mandible; anterior tentorial pits indistinct (Fig 7B); length of malar space 0.1 times basal width of mandible; mandible 1.9 times as long as wide, largely smooth, with a lamella ventrally and dorsally, with medium-sized carina connected to third tooth.

Mesosoma. Length of mesosoma 1.3 times its height; pronotum without pronope; antescutal depression distinct and finely crenulate laterally; side of pronotum smooth; epicnemial area smooth; precoxal sulcus complete; remainder of mesopleuron smooth; episternal scrobe elliptical; pleural sulcus smooth, except for some crenulae medially and ventrally; mesosternal sulcus wide and with 3 short crenulae; metapleuron largely smooth, except for a few carinae ventrally; notauli only anteriorly impressed, with strong lateral carina and largely absent on disc; medio-posterior depression of mesoscutum small and nearly round (Fig 7C); mesoscutum smooth and largely glabrous and its lateral carina absent in front of tegulae; scutellar sulcus moderately deep and with one carina, as long as scutellum; scutellum in lateral view rather flat; metanotum slightly lamelliform protruding dorsally in lateral view; surface of propodeum smooth except for a medium-sized median carina, rather short lamelliform costulae and a narrow barrel-shaped areola (Fig 7D), its dorsal corners hardly protruding in lateral view and lateral carina of propodeum wide.

Wings. Pterostigma narrow triangular (Fig 7E); vein r 0.25 times width of pterostigma, issued from middle of pterostigma and vertical; r:3-SR:SR1 = 5:80:150; SR1 straight; cu-a obsolescent, postfurcal; 1-CU1:2-CU1 = 5:30; 2-SR:3-SR:r-m = 36:80:15; m-cu far antefurcal (2-SR+M 0.3 times as long as m-cu), forming an almost straight line with 2-SR+M, converging to 1-M. Hind wing: M+CU + 1-M:1r-m = 80:19; cu-a obsolescent.

Legs. Hind coxa smooth and elongate, rounded basally; tarsal claws slender medially; length of femur, tibia and basitarsus of hind leg 5.0, 11.0 and 3.0 times their width, respectively; hind femur, tibia and tarsus with long erect setae.

Metasoma. Length of first tergite twice its apical width, its surface largely smooth but coarsely rugose medially and laterally lamelliform, its dorsal carinae nearly complete; laterope present; dorsope long and area laterally lamelliform; remainder of metasoma smooth and depressed; length of setose part of ovipositor sheath 0.37 times fore wing and about as long as hind tibia and with a few long setae; ovipositor sheath parallel-sided.

Colour. Dark orange; but following parts brown: head, 5^th^-17^th^ antennal articles (and 18^th^-21^rd^articles whitish), anterior part of mesoscutum, ventral half of mesopleuron and metasoma (except first tergite); wing membrane slightly infuscate; pterostigma (but apically rather pale) and veins brown.

Variation. Not much in the few specimens at hand.

#### Host

Collected in banana traps infested by unidentified Drosophilidae.

#### Distribution

China (Yunnan), South Korea.

#### Diagnostic remarks

The new species is similar to *A*. *leveri* (Nixon). It differs by having the second submarginal cell of fore wing wide basally (narrow basally *A*. *leveri*); medio-posterior pit of mesoscutum round or nearly so (short elliptical); yellow scapus slightly or not contrasting with yellowish brown to brown vertex (yellow scapus contrasting with dark brown vertex) and length of setose part of ovipositor sheath 1.0–1.1 times hind tibia and 0.4 times fore wing (1.1–1.2 times and 0.5 times, respectively).

### Molecular analysis

**Fig 8:**
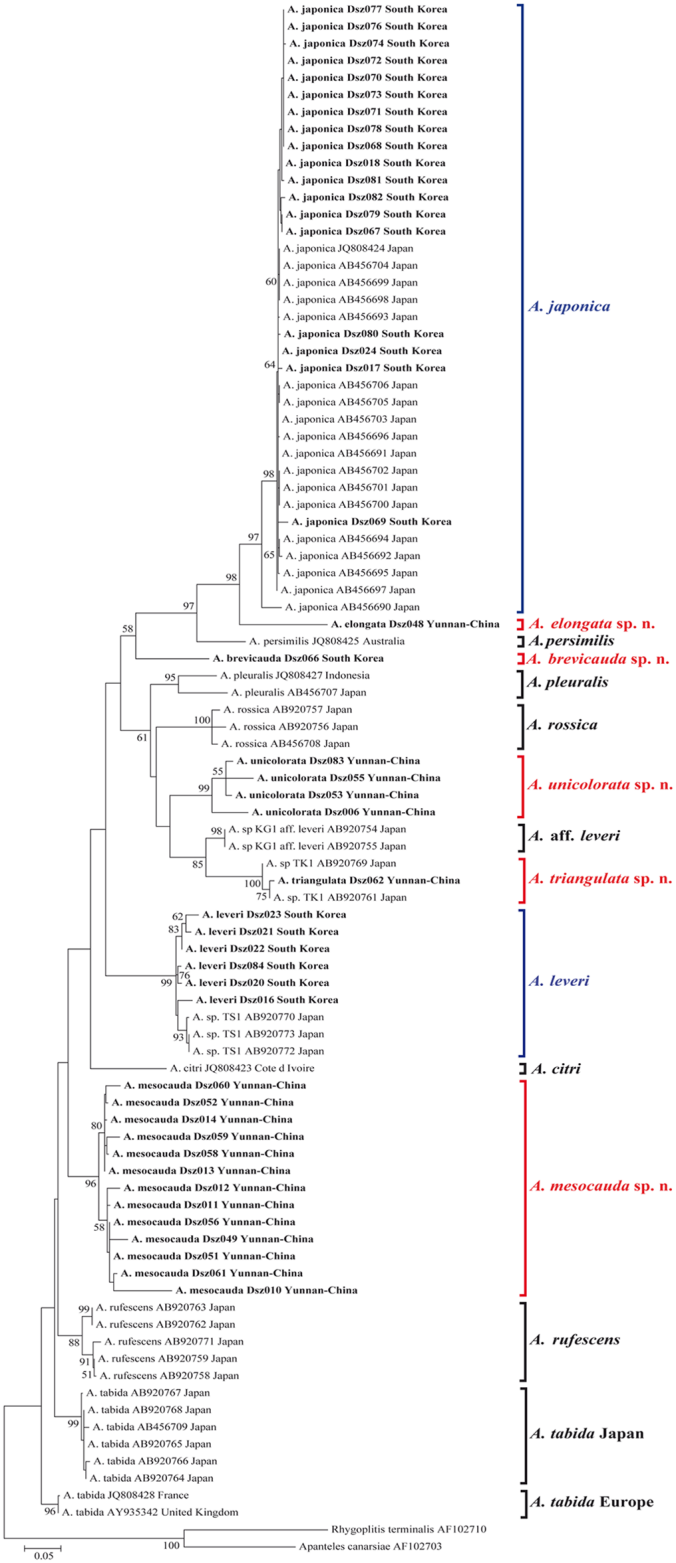
Maximum Likelihood tree based on partial nucleotide sequence of COI gene. Bootstrap values ≥50% are indicated on the branches. Scale bar indicates the number of substitution per site. Asobara individuals sampled and sequenced in this study are in bold. On the right side of the tree, the names of Asobara species found in this work are in red for the new species herein described or in blue for the species already known.

Trimmed COI sequences resulted in a fragment of size ranging between 410 and 640 bp. Alignment was straightforward with no frame shifts, nonsense codons, insertions or deletions identified in any sequence. ML and NJ analyses produced trees of almost identical topology, well supporting the distinction between the seven morphospecies collected in China and South Korea in this work, each one appearing as a different lineage on the tree (Fig 8). Overall, at least 15 different species were supported by phylogenetic analysis. The average uncorrected COI *p*-distance between species ranged from 5.9% and 20.2%. The average uncorrected COI *p*-distance within species ranged from 0% to 4.7%, except in *A*. *pleuralis* (8.7%) (S1 Table. Uncorrected p-distance (number of base differences per site) between COI sequences of *Asobara* species.; S2 Table. Intraspecific uncorrected p-distances (number of base differences per site) from averaging over all COI sequence pairs within each *Asobara* species.; S3 Table. Interspecific uncorrected p-distances (number of base differences per site) from averaging over all COI sequence pairs between *Asobara* species. Standard error estimates are shown above the diagonal.).

## Discussion

*Asobara* is a rather large genus with nearly 40 valid species known worldwide (some of the Neotropical species may not belong in *Asobara* [24]. *Asobara* species are mainly larval koinobiont endoparasitoids of flies in the family Drosophilidae and Sepsidae living in decaying organic matter, especially fruits and leaves. Some species with widened ovipositor sheath have been reared as parasitoids of Tephritidae in fruits [25]. The genus is currently revised by the author CvA and colleagues, and many new species are waiting to be described. In this work, using morphological and molecular analyses, we found a considerable diversity of *Asobara* parasitoids in the Yunnan Province of China and in South Korea. Out of the four species collected in China, all are new to science and are formally described as *A*. *elongata*, *A*. *mesocauda*, *A*. *unicolorata* and *A*. *triangulata*. Out of the three species collected in South Korea, only one is new to science and is formally described as *A*. *brevicauda*. The other two species, *A*. *japonica* and *A*. *leveri*, are well known for the Asian continent [27]. However, a further cryptic diversity may be hidden within the new species here described. Because of the high variability of COI sequence observed in some lineages, as for example in *A*. *unicolorata* (average uncorrected *p*-distance 4.7%, range 1.2–8.7%) and *A*. *mesocauda* (average uncorrected *p*-distance 3.3%, range 0–9.7%), it is likely that each of the two morphospecies here described represents a complex of cryptic species whose identity and discriminating characters can be revealed by further investigations and only after the collection of large numbers of individuals necessary to set up a multivariate morphometric analyses [28,29]. Nonetheless, inter and intraspecific genetic distances (as reported in S1 Table. Uncorrected p-distance (number of base differences per site) between COI sequences of *Asobara* species.; S2 Table. Intraspecific uncorrected p-distances (number of base differences per site) from averaging over all COI sequence pairs within each *Asobara* species.; S3 Table. Interspecific uncorrected p-distances (number of base differences per site) from averaging over all COI sequence pairs between *Asobara* species. Standard error estimates are shown above the diagonal) clearly indicate the correct identification of different entities further confirmed by the phylogenetical analysis (Fig 8).

*Asobara triangulata* shows identical COI sequences to individuals of *A*. sp. TK1 found in Tokyo but also in the south-western isle of Fukue Japan, not distant from the South Korean coast [27], suggesting a large distribution range of this species in South-eastern Asia. Similarly, COI sequences of *A*. *leveri* here collected in South Korea group with sequences of individuals of *A*. sp. TS1, collected in the small south-western island of Japan, Tsushima and Fukue-jima, very close to South Korean coast [27], strongly supporting conspecificity. Finally, as already reported [27], we found that *A*. *tabida* from Japan forms a lineage distantly related to *A*. *tabida* from Europe, indicating a status of separate species.

Despite the pest status of *D*. *suzukii* and the absence of effective natural enemies in the invaded areas of Europe and North America [10–13] a deep knowledge of parasitoids of *D*. *suzukii* in its native area, the Southeastern Asia, is still lacking, limiting the opportunity to implement biological control programmes. Most information is from Japan, where a few parasitoid species were found to parasitize *D*. *suzukii* in the field, including *Ganaspis xanthopoda* (Ashmead) and *Leptopilina japonica* Novkovic & Kimura (Hymenoptera: Figitidae), *Trichopria* sp.(Hymenoptera, Diapriidae), and six species of *Asobara*. The most common resulted *A*. *japonica*, while *A*. *rossica*, *A*. *rufescens*, *A*. *tabida* and two unidentified species completed the *Asobara* complex [30,31,27]. However, the parasitisazion rate observed in the field was very low and in general all this species appeared able to parasitise a wide range of Drosiphilid species, overall resulting of limited interest as biological control agents. Only one species, *Asobara* sp. TK1, emerged only from *D*. *suzukii* [27]. On the basis of our results, this species should be considered within the concept of *A*. *triangulata* sp. nov. However, we cannot confirm the specificity to *D*. *suzukii* because the single individual here examined was collected in China with banana-baited trap colonized by different species of drosophilids, including *D*. *suzukii*. In this work, two *Asobara* species could be associated with *D*. *suzukii* as collected from berries of wild *Rubus* sp. infested by the fruit fly (*A*. *japonica*) or from fruit-baited trap infested with *D*. *suzukii* before placement in the field (*A*. *leveri*). Our findings, far from indicating a silver bullet in the control of *D*. *suzukii*, present new species for quarantine evaluation as biocontrol agents and point out the importance of further collections in the native area of *D*. *suzukii* where biodiversity of its parasitoids appears to be extremely wide.

## Supporting Information

S1 Table Uncorrected p-distance (number of base differences per site) between COI sequences of *Asobara* species. (XLS)

S2 Table Intraspecific uncorrected p-distances (number of base differences per site) from averaging over all COI sequence pairs within each *Asobara* species. (XLS)

S3 Table Interspecific uncorrected p-distances (number of base differences per site) from averaging over all COI sequence pairs between *Asobara* species. Standard error estimates are shown above the diagonal.
(XLS)
